# Neuroprotective effects of flavonoids on hypoxia-, glutamate-, and oxidative stress–induced retinal ganglion cell death

**Published:** 2011-07-02

**Authors:** Mao Nakayama, Makoto Aihara, Yi-Ning Chen, Makoto Araie, Kaori Tomita-Yokotani, Tsukasa Iwashina

**Affiliations:** 1 Department of Ophthalmology, University of Tokyo School of Medicine, Tokyo, Japan; 2 Graduate school of Life and Environmental Sciences, University of Tsukuba, Tsukuba, Ibaraki, Japan; 3 Department of Botany, National Museum of Nature and Science, Ibaraki, Japan

## Abstract

**Purpose:**

This study was conducted to investigate the effect of flavonoids, a major family of antioxidants contained in foods, on retinal ganglion cell (RGC) death induced by hypoxia, excessive glutamate levels, and oxidative stress. Moreover, to assess the structure-activity relationships of flavonoids, three types of flavonoids with different numbers of hydroxyl groups and varieties of sugar chains were studied.

**Methods:**

Three kinds of flavonoids—nicotiflorin, rutin, and quercitrin—were used. The death of neonatal rat purified RGCs was induced by hypoxic conditions (5% O_2_, 5% CO_2_, 37 °C) for 12 h, 25 µM glutamate over three days, or oxidative stress by depleting antioxidants from the medium for 24 h. RGC survival rates were calculated under each condition and compared with vehicle cultures. Modification of cell death signaling after stress-induced apoptosis and necrosis by flavonoids was assessed using caspase-3 and calpain immunoreactivity assays.

**Results:**

Under hypoxic and glutamate stress, both nicotiflorin and rutin significantly increased the RGC survival rate at 1 nM or higher, while quercitrin increased it at 100 nM or higher. Under oxidative stress, nicotiflorin, rutin, and quercitrin also significantly increased the RGC survival rate at 1 nM, 0.1 nM, and 100 nM or higher, respectively. Rutin significantly inhibited the induction of caspase-3 under both hypoxia and excessive glutamate stress, as well as blocking the induction of calpain during oxidative stress.

**Conclusions:**

Nicotiflorin and rutin showed neuroprotective effects on hypoxia-, glutamate- or oxidative stress–induced RGC death at concentrations of 1 nM or higher. The presence of a specific sugar side chain (rutinoside) may enhance neuroprotective activity.

## Introduction

Hypoxia, glutamate, and oxidative stress are important factors in ocular disease states affecting retinal ganglion cells (RGCs). Retinal ischemic and inflammatory diseases such as retinal vascular occlusion or diabetic retinopathy elicit hypoxic and/or oxidative stress–induced RGC death [1-3]. Likewise, RGC death in glaucoma is believed to be induced by apoptotic mechanisms triggered by multiple stimuli, including ischemia, oxidative stress, and elevation of glutamate levels [4,5]. Numerous studies have demonstrated that excessive glutamate induces RGC death in vitro and in vivo [6], and that the glutamate receptor antagonists a non-competitive antagonist of the N-methyl-d-aspartate (NMDA) receptor, (MK801) or memantine can ameliorate RGC death caused by elevated intraocular pressure [7-11]. Oxidative stress induced either by increased levels of reactive oxygen species (ROS) or mitochondrial dysfunction is also implicated in glaucomatous, ischemic, and hereditary optic neuropathies [12,13].

Flavonoids comprise a large family of plant-derived compounds widely distributed in fruits and vegetables [14,15]. There is growing evidence from human nutrition studies that the absorption and bioavailability of specific flavonoids is much higher than originally believed [15,16]. Flavonoids are believed to exert protective as well as beneficial effects on multiple disease states, including cancer, cardiovascular disease, and neurodegenerative disorders [15,17,18]. These physiologic benefits of flavonoids are generally thought to be derived from their antioxidant and free radical–scavenging properties [19]. Accordingly, flavonoids may also have therapeutic potential in ocular diseases. However, only four studies describing the potential effects of flavonoids on RGC death using RGC-5 transgenic cell lines or in vivo rodent models have been reported [20-23].

In the current study, we selected three types of flavonoid compounds—kaempferol 3-*O*-rutinoside (nicotiflorin), quercetin 3-*O*-rutinoside (rutin), and quercetin 3-*O*- rhamnoside (quercitrin)—from more than 7,000 known flavonoids, and investigated their neuroprotective potential using rat primary-isolated RGCs cultured under three kinds of stress conditions: hypoxia, excessive glutamate levels, and oxidative stress. Since these three compounds have quite similar structures, excluding the number of hydroxyl groups and sugar side chains, the structure-activity relationships for neuroprotective activity were also considered.

## Methods

### Animals

Six- to eight-day-old Wistar rats were purchased from Saitama Laboratory Animal Supply Inc. (Saitama, Japan). All animal experiments were conducted with approval from the Animal Care and Use Committee and under the guidelines of the ARVO Statement for the Use of Animals in Ophthalmic and Vision Research.

### Materials

Cell culture reagents were obtained from Gibco (Grand Island, NY). A cell-dissection kit of papain was obtained from Worthington Biochemical (Lakewood, NJ). Calcein-AM was obtained from Sigma (St. Louis, MO). Poly-L-lysine, BSA, L-glutamine, and recombinant neurotrophic factors (human brain-derived neurotrophic factor and rat ciliary neurotrophic factor) were obtained from Sigma. Monoclonal antibodies (OX-41) against rat macrophages and monoclonal antibodies (OX-7) against rat and mouse Thy-1.1 were obtained from Chemicon (Temecula, CA). B-27 supplement minus antioxidants (AO-) was obtained from Gibco. The origins of the flavonoids were as follows: kaempferol 3-*O*- rutinoside (nicotiflorin) was purchased from Extrasynthese (Genay, France); quercetin 3-*O*-rhamnoside (quercitrin) was from Synthese (Lab Sarget, Merignac, France); and quercetin 3- *O*-rutinoside (rutin) was purified by one of the authors, Tsukasa Iwashina, from the flowers of *Astrophytum ornatum* (Britton & Rose) [24]. All flavonoids were dissolved in 100% ethanol and diluted into a 10 mM solution with sterilized distilled water. The 10 mM flavonoid stocks were filtered using 0.45 mm filters (DISMIC, Toyo Roshi, Tokyo, Japan) for sterilization and stored at 4 °C in a 5 ml sterile tube (Greiner Bio-One, Frickenhausen, Germany) until use. For the vehicle solutions, the same dilution process was used. The chemical structures of these three compounds are indicated. Unless noted, all other reagents were obtained from Invitrogen (Carlsbad, CA).

### Purification and culture of rat retinal ganglion cells

RGCs were purified by a two-step immunopanning procedure, as described previously [25]. Briefly, the dissociated retinal cells from eight-day-old Wister rats were incubated in flasks (Nunc A/S, Roskilde, Denmark), coated with an antirat macrophage monoclonal antibody (1:50) to exclude macrophages, and then incubated in tubes (Corning, Acton, MA) coated with an antirat Thy1.1 monoclonal antibody (1:300). RGCs adherent to the tubes were collected by centrifugation at 60× g for 5 min and seeded on 13 mm glass coverslips in a 24-well plate that had been coated with 50 µg/ml poly-L-lysine (Sigma) and 1 µg/ml laminin (Invitrogen). Purified RGCs were plated at a density of approximately 1,000 cells per well. RGCs were cultured in serum-free B27 complete medium containing neurobasal medium (Invitrogen) with 1 mM L-glutamine (Sigma), B27 supplement (Invitrogen), 40 ng/ml human recombinant brain-derived neurotrophic factor, 40 ng/ml rat recombinant ciliary neurotrophic factor, 10 µM forskolin (Sigma), 100 U/ml penicillin, and 100 µg/ml streptomycin. Plates were incubated in a tissue culture incubator with a humidified atmosphere containing 5% CO_2_ and 95% air at 37 °C for 3 days.

### Induction of hypoxia, glutamate, and oxidative stress

After culturing for three days, RGC death was induced by the following three conditions. For hypoxia, the plates were transferred to a controlled-atmosphere incubator and incubated for 12 h, during which oxygen levels were reduced (hypoxic condition: 5% CO_2_, 90% N_2_, and 5% O_2_ mixture) [26]. For excessive glutamate stress, 25 µM glutamate was added to the serum-free B27 neurobasal medium containing RGCs at a final concentration, and incubated for another three days under normoxic conditions (5% CO_2_, 95% air, at 37 °C) [27]. For oxidative stress, glass coverslips adhered with RGCs were moved to neurobasal medium containing B27 with diminished antioxidants (AO−) including reduced glutathione, vitamin E, vitamin E acetate, catalase, and superoxide dismutase (B27 supplement minus AO 50×; Invitrogen), and incubated for 24 h under normoxic conditions [28].

### Evaluation of the protective effects of flavonoids

Nicotiflorin and rutin were added at final concentrations of 0.1 nM, 1 nM, and 10 nM, and quercitrin at 10 nM, 100 nM, and 1 µM. Culture medium with flavonoids were made by a serial dilution with B27 neurobasal medium. In the present study, a surviving RGC was defined as a cell with a calcein-AM-stained cell body and a process extending at least three cell diameters from the cell body [25,26,29]. All surviving RGCs on each glass coverslip were counted at 200× magnification using an inverted fluorescence microscope (Nikon Eclipse TE300). In each experiment, the number of RGCs cultured in nonstress conditions (control group) was set at 100%. In the control culture, about 1,000 cells were counted in one assay. We repeated at least eight assays for each experiment. The percentage of surviving RGCs after stress was calculated and normalized to the control group without stress and flavonoid treatment. Each percentage is expressed in the text and figures as the mean±standard deviation (SD).

### Assessment of caspase-3 and calpain activity

In our previous studies, we determined the cell death patterns of purified RGCs where death was induced by hypoxia, glutamate, and oxidative stress under our culture conditions, and found that both hypoxia- and glutamate-induced death mainly involved mitochondrial-dependent and Bax-mediated apoptosis [27], while oxidative stress mainly induced necrotic cell death [28]. To investigate the effect of flavonoids on apoptotic and necrotic cell death signaling, we measured the activity of caspase-3, one of the main proteases related to apoptosis, under stress in the presence or absence of 10 nM rutin. To investigate the effect of flavonoids on necrotic cell death signaling, we measured the activity of calpain, one of the main proteases related to necrosis, under stress in the presence or absence of 10 nM rutin. Caspase-3 activity was determined by a Caspase-3/cpp32 Colorimetric Assay Kit (Bio Vision, Mountain View, CA). Calpain activity was determined by a Calpain Activity Assay Kit (Bio Vision, Mountain View, CA). Both activities were determined by measures of absorbance at the 405 nm wavelength.

### Statistical analyses

The Student *t* test or one-way ANOVA followed by Dunnett’s test were used in this study. All data are indicated as mean±SD.

## Results

### Effect of flavonoids on hypoxia-induced retinal ganglion cell death

**Figure 1:**
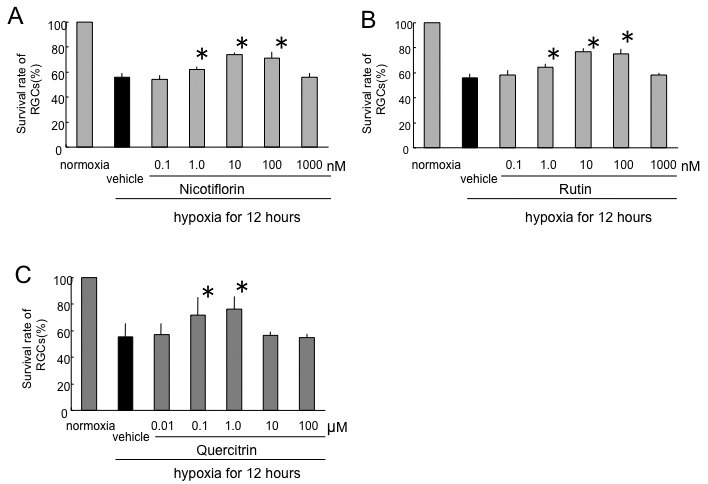
Effects of nicotiflorin (**A**), rutin (**B**), and quercitrin (**C**) against hypoxia-induced retinal ganglion cell death. Each bar represents mean±standard deviation (SD; n=8). The asterisks indicate p<0.05 versus hypoxia vehicle (black bar) by Dunnett’s tests.

After 12 h of hypoxia stress, the RGC survival rate without nicotiflorin was reduced to 56.0±3.1%. The RGC survival rates of the nicotiflorin-treated group were 54.3±3.2%, 62.3±1.9%, 73.8±2.0%, 71.4±4.9%, and 56.1±3.2% at final concentrations of 0.1 nM, 1 nM, 10 nM, 100 nM, and 1 µM, respectively (Figure 1A). The RGC survival rate without rutin was reduced to 56.0±3.1%. The RGC survival rates of the rutin-treated group were 58.4±3.5%, 64.3±2.8%, 76.7±3.2%, 75.0±3.9%, and 58.4±1.6% at final concentrations of 0.1 nM, 1 nM, 10 nM, 100 nM, and 1 µM, respectively (Figure 1B). The RGC survival rate without quercitrin was reduced to 55.5±10.0%. The RGC survival rates of the quercitrin-treated group were 56.9±8.9%, 72.0±13.4%, 76.0±9.9%, 56.4±2.7%, and 54.6±3.0% at final concentrations of 10 nM, 100 nM, 1 µM, 10 µM, and 100 µM, respectively (Figure 1C). The addition of nicotiflorin and rutin at 1 nM, 10 nM, and 100 nM, and quercitrin at 100 nM and 1 µM, significantly increased RGC viability (p<0.05 versus vehicle by Dunnett’s test, n=8; Figure 1).

### Effect of flavonoids on glutamate-induced retinal ganglion cell death

**Figure 2:**
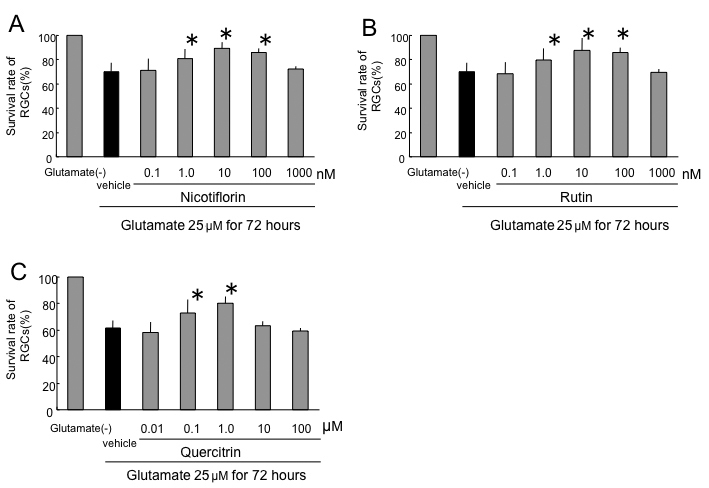
Effects of nicotiflorin (**A**), rutin (**B**), and quercitrin (**C**) against 25 µM glutamate-induced retinal ganglion cell death. Each bar represents mean±standard deviation (SD; n=8). The asterisks indicate p<0.05 versus glutamate vehicle (black bar) by Dunnett’s tests.

After 72 h of glutamate stress, the RGC survival rate without nicotiflorin was reduced to 69.8±7.4%. The RGC survival rates in the nicotiflorin-treated group were 71.4±9.2%, 80.9±7.8%, 89.1±5.2%, 85.7±3.4%, and 72.2±2.6% at final concentrations of 0.1 nM, 1 nM, 10 nM, 100 nM, and 1 µM, respectively (Figure 2A). The RGC survival rate without rutin was reduced to 69.8±7.4%. The RGC survival rates of the rutin-treated group were 68.5±9.6%, 79.5±9.5%, 87.6±9.9%, 85.8±4.1%, and 69.5±2.9% at final concentrations of 0.1 nM, 1 nM, 10 nM, 100 nM, and 1 µM, respectively (Figure 2B). The RGC survival rate without quercitrin was reduced to 61.8±5.4%. The RGC survival rates of the quercitrin-treated group were 58.2±8.0%, 73.0±9.8%, 80.0±5.5%, 63.2±3.3%, and 59.9±1.8% at final concentrations of 10 nM, 100 nM, 1 µM, 10 µM, and 100 µM, respectively (Figure 2C). The addition of nicotiflorin and rutin at 1 nM, 10 nM, and 100 nM, and quercitrin at 100 nM and 1 µM, significantly increased RGC viability (p<0.05 versus vehicle by Dunnett’s test, n=8; Figure 2).

### Effect of flavonoids on oxidative stress–induced retinal ganglion cell death

**Figure 3:**
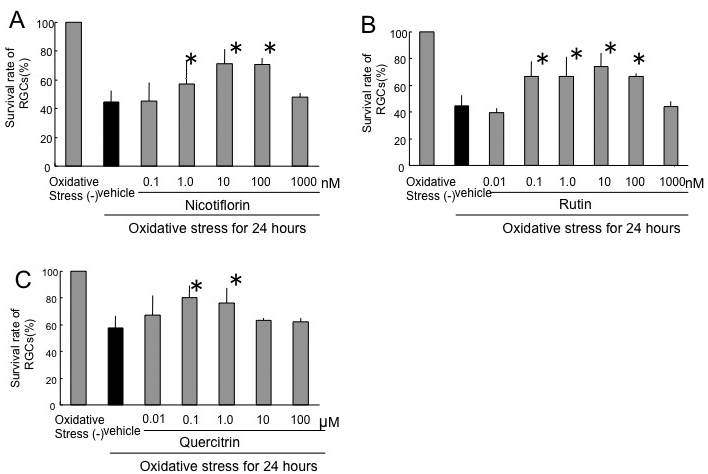
Effects of nicotiflorin (**A**), rutin (**B**), and quercitrin (**C**) against oxidative stress–induced retinal ganglion cell death. Each bar represents mean±SD (n=8). The asterisks indicate p<0.05 versus oxidative stress vehicle (black bar) by Dunnett’s tests.

After 24 h of oxidative stress, the RGC survival rate without nicotiflorin was reduced to 44.4±8.1%. The RGC survival rates of the nicotiflorin-treated group were 45.4±12.6%, 57.3±15.8%, 71.2±9.9%, 70.8±4.1%, and 48.1±2.9% at final concentrations of 0.1 nM, 1 nM, 10 nM, 100 nM, and 1 µM, respectively (Figure 3A). The RGC survival rate without rutin was reduced to 44.4±8.1%. The RGC survival rates of the rutin-treated group were 39.9±3.4%, 66.6±11.2%, 66.7±14.5%, 74.2±10%, 66.4±2.3%, and 44.0±4.2% at final concentrations of 0.01 nM, 0.1 nM, 1.0 nM, 10 nM, 100 nM, and 1 µM, respectively (Figure 3B). The RGC survival rate without quercitrin was reduced to 57.7±8.7%. The RGC survival rates of the quercitrin-treated group were 67.4±14.7%, 80.0±9.1%, 76.5±10.8%, 63.1±1.9%, and 61.9±3.2% at final concentrations of 10 nM, 100 nM, 1 µM, 10 µM, and 100 µM, respectively (Figure 3C). The addition of nicotiflorin at 1 nM, 10 nM, and 100 nM; rutin at 0.1 nM, 1 nM, 10 nM, and 100 nM; and quercitrin at 100 nM and 1 µM significantly increased RGC viability (p<0.05 versus vehicle by Dunnett’s test, n=8; Figure 3).

### Caspase-3 activity in stress-induced retinal ganglion cell death

**Figure 4:**
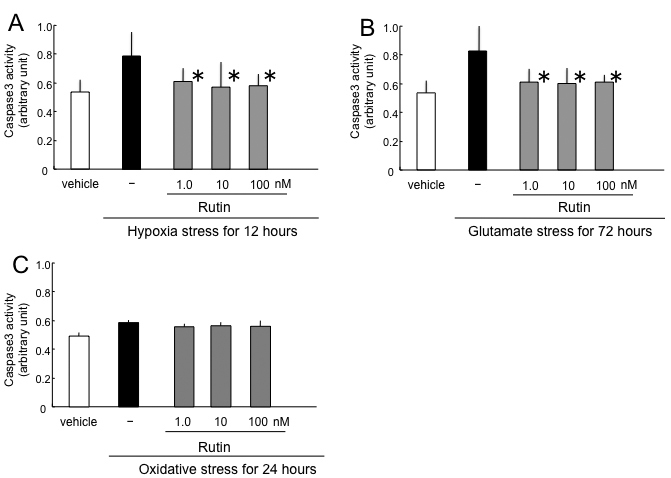
Effect of rutin on caspase-3 activity induced by hypoxia (**A**), glutamate (**B**), and oxidative stress (**C**). The asterisks indicate p<0.05 by Dunnett’s tests compared to stress only (black bar).

After 12 h of hypoxia, caspase-3 activity in RGCs without rutin was 0.80±0.14, whereas caspase-3 activity without hypoxia was 0.55±0.11. Caspase-3 activities with rutin were 0.62±0.1, 0.58±0.21, and 0.59±0.09 at final concentrations of 1 nM, 10 nM, and 100 nM, respectively (arbitrary units). Rutin showed a significant reduction of caspase-3 activity under hypoxia (p<0.05 by Dunnett’s test compared to hypoxia stress only, n=6; Figure 4A).

After 72 h of glutamate stress, caspase-3 activity in RGCs without rutin was 0.84±0.25, whereas caspase-3 activity without glutamate was 0.54±0.14. Caspase-3 activities in RGCs with rutin were 0.63±0.13, 0.61±0.15, and 0.62±0.09 at final concentrations of 1 nM, 10 nM, and 100 nM, respectively (arbitrary units). Rutin showed a significant reduction of caspase-3 activity by glutamate stress (p<0.05 by Dunnett’s test compared to glutamate stress only, n=6; Figure 4B).

After 24 h of oxidative stress, caspase-3 activity in RGCs without rutin was 0.61±0.02, whereas caspase-3 activity without oxidative stress was 0.51±0.03. Caspase-3 activities in RGCs with rutin were 0.58±0.02, 0.59±0.02, and 0.59±0.04 at final concentrations of 1 nM, 10 nM, and 100 nM, respectively (arbitrary units). There was no significant change of caspase-3 activity by oxidative stress (n=6; Figure 4C).

### Calpain activity in oxidative stress–induced retinal ganglion cell death

**Figure 5:**
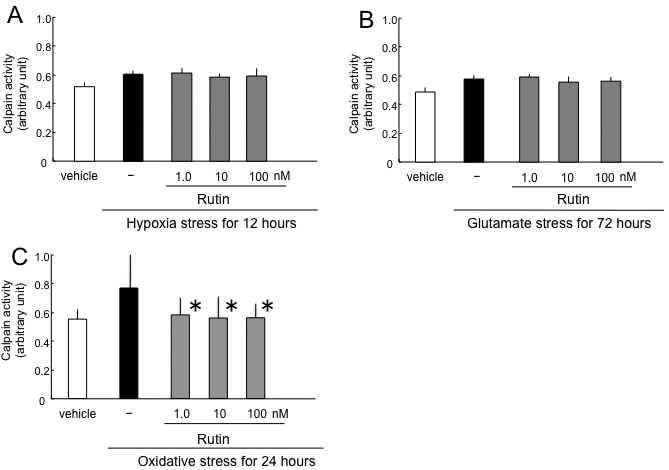
Effect of rutin on calpain activity induced by hypoxia (**A**), glutamate (**B**), and oxidative stress (**C**). The asterisks indicate p<0.05 by Dunnett’s tests compared to stress only (black bar).

After 12 h of hypoxia, calpain activity in RGCs without rutin was 0.59±0.03, whereas caspase-3 activity without hypoxia was 0.51±0.03. Calpain activities in RGCs with rutin were 0.60±0.4, 0.58±0.02, and 0.58±0.05 at final concentrations of 1 nM, 10 nM, and 100 nM, respectively (arbitrary units). There was no significant change of calpain activity under hypoxia (n=6; Figure 5A).

After 72 h of glutamate stress, calpain activity in RGCs without rutin was 0.60±0.03, whereas caspase-3 activity without glutamate was 0.51±0.03. Calpain activities in RGCs with rutin were 0.62±0.02, 0.58±0.04, and 0.59±0.03 at final concentrations of 1 nM, 10 nM, and 100 nM, respectively (arbitrary units). There was no significant change of calpain activity under hypoxia (n=6; Figure 5B).

After 24 h of oxidative stress, calpain activity in RGCs without rutin was 0.78±0.19, whereas caspase-3 activity without oxidative stress was 0.56±0.14. Calpain activities in RGCs with rutin were 0.59±0.13, 0.58±0.23, 0.57±0.13 at final concentrations of 1 nM, 10 nM, and 100 nM, respectively (arbitrary units). Rutin showed significant reduction of calpain activity by oxidative stress (p<0.05 by Dunnett’s test compared to oxidative stress only, n=6; Figure 5C).

## Discussion

Our results using rat purified primary RGC cultures first indicated that flavonoids had a strong neuroprotective effect against RGC death induced by hypoxia, excessive glutamate levels, and oxidative stress. Experiments using rutin indicated that flavonoids could influence both apoptotic and necrotic RGC death at concentrations as low as 1 nM. Moreover, the structure-activity relationships among the three flavonoids indicated that the rutinoside sugar chain might be important in exerting neuroprotective activity.

In this study, we only selected three kinds of flavonols, which had similar chemical structures. Although many other number of flavonoids should be assessed to identify more details of the structure-activity relationships for various stresses, there was a limitation in obtaining the necessary number of purified rat RGCs for our biologic assays. The RGC-5 cell line is useful for a large amount of cells, but the nature of the cells is quite different for the primary RGCs [30]. A more easy and efficient assay system to screen and select the effective drugs should be developed in future.

### Effectiveness at a low dose

In the current study, a rat purified RGC culture system was used as an experimental model to survey the neuroprotective effects of flavonoids on RGC death induced by three kinds of stresses. The effective doses of two flavonoids were very low (~1 nM). In our previous studies using the same hypoxic culture system, the effective doses of lomerizine, a Ca^2+^ channel blocker, and betaxolol, an antiglaucoma drug with weak Ca^2+^ channel blocking activity, were 10 and 100 nM, respectively [26,27]. Thus, flavonoids may be more effective neuroprotective agents than Ca^2+^ channel blockers, which are well known neuroprotectants for ischemia-induced cell death [31].

In previous reports about flavonoids using the RGC-5 cell line, the effective doses (over 10 µM) were quite a bit higher than our results, although the examined flavonoids and the conditions to induce RGC-5 cell death were different from those used in our experiments [20-23]. Another reason for this discrepancy could be the use of an immortalized RGC-5 cell line that may be more tolerant than primarily isolated RGCs against various stresses. Additionally, a recent report indicated that RGC-5 had different characteristics from the original cells in terms of missing RGC cell markers and conventionally expressed proteins [30]. Two previous reports using in vivo rodent models indicated that intraperitoneal injection of the flavonoid baicalin (15 mg/kg) or catechin (25 mg/kg) was neuroprotective, but the actual tissue concentrations of these substances were not measured [20,23]. Based on the current results, rutin and nicotiflorin may deserve further study to validate their in vivo neuroprotective potential.

### Structure-activity relationships conferring neuroprotection

**Figure 6:**
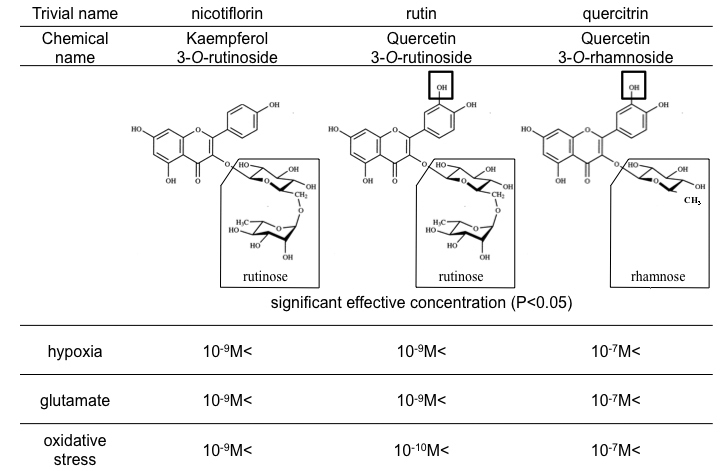
Structures and effective concentrations of nicotiflorin, rutin, and quercitrin against the three stresses. Note the difference in side chains enclosed by rectangles. P-value<0.05 by calculated in Dunnett's tests compared to stress only. The result showed that neuroprotective concentration of quercitrin is different with nicotiflorin and rutin. It is shown that different structures of changing side chain induced different concentrations of neuroprotection.

So far, there have been no reports comparing flavonoid structure-activity relationships. This is the first study to indicate the differences in biologic activities among three kinds of flavonoids with a similar chemical structure (shown in Figure 6). Although the precise mechanism of action remains unclear, the beneficial activity of flavonoids is generally attributed to their antioxidative efficacy [19,32,33]. The antioxidant capacity of flavonoids depends on the arrangement of functional groups surrounding the flavonol nucleus, which may directly affect glutathione metabolism, antioxidant capacity, or the ability to maintain low Ca^2+^ levels despite high levels of ROS [14,19]. In this study, both nicotiflorin and rutin displayed neuroprotective effects at concentrations of 1 nM or higher, whereas quercitrin was only effective at 100 nM or higher. Comparison of the chemical structures of the three flavonoids (Figure 6) indicates that the sugar side chain may be important for neuroprotective activity, since quercitrin (with the same quercetin nucleus as rutin, but with a rhamnoside sugar chain in lieu of a rutinoside sugar chain) showed about 100 times weaker efficacy compared to rutin. In comparison, the numbers of hydroxyl groups in the flavonol nucleus may be unrelated to the antioxidative activity, because nicotiflorin and rutin showed similar neuroprotective activity despite possessing a different number of hydroxyl groups (Figure 6). This novel finding regarding flavonoid structure-activity relationships may be useful in the development of more effective flavonoid antioxidants.

### Unknown mechanism of action

Oxidative stress has been implicated in many types of ocular diseases. Several previous reports have shown that RGC damage under oxidative stress is mainly linked with increased intracellular ROS and calcium influx [22,28]. During ischemia/reperfusion in ocular vascular diseases, tissue reoxygenation also induced an overproduction of ROS [1-3]. Furthermore, some studies indicated that excitatory transmitter amino acids such as glutamate were released under oxidative and hypoxic stresses [27,34-36]. Therefore, one of the reasons that flavonoids were effective not only for oxidative stress but also for hypoxic or excessive glutamate stress might be due to a partial involvement of oxidative stress factors in hypoxia- or glutamate-induced cell death. On the other hand, it is also possible that flavonoids have effects other than antioxidation during hypoxia- or glutamate-induced cell death.

Rutin, as a representative of the three flavonoids, caused a significant inhibition of both apoptotic and necrotic pathways in RGCs. In this study, we investigated the effect of rutin on the activities of caspase-3 and calpain, which are the representative proteases in apoptotic and necrotic pathways, respectively. As a result, rutin suppressed these activities, but there was no dose-dependency. These data suggest that other protective signals in addition to caspase-3 or calpain may be present. There is a limitation to investigating the signaling molecules related to apoptosis and necrosis using primary cultures of RGCs, because a large number of primary cells is required for biochemical assays targeting various death signals. Further experiments are required to clarify the mechanism of these flavonols.

### Clinical relevance of flavonoids

Nicotiflorin and rutin, with only one hydroxyl group difference between them structurally, are contained mainly in fruits and vegetables. Quercitrin, with a different sugar side chain than rutin, is contained mainly in tea and some medicinal plants. The beneficial effects of dietary plant substances containing these flavonoids has been recorded since ancient times and passed on for generations. Buckwheat and tartary buckwheat, which are commonly ingested in Japan and other Asian countries, are both rich in rutin; amazingly, rutin accounts for as high as 1% of the total weight of buckwheat and tartary buckwheat [37,38]. A few reports have indicated that repeated intake of several hundred milligrams of rutin resulted in a plasma concentration of 100 nM or higher [39-41]. Moreover, it has been reported that flavonoids can penetrate into the central nervous system through the blood-brain barrier [42]. Currently, their multitudinous pharmacological effects, such as their antioxidant capacity and anticancer effects, are being scientifically and clinically evaluated in the hopes of reducing the risk of cardiovascular disease and/or inhibiting tumor angiogenesis [17,37,43-48]. Our current results also suggest that flavonoids have the potential to be clinically useful for hypoxia-, glutamate-, or oxidative stress–related RGC morbidities.

### Conclusion

In summary, we studied the neuroprotective effects of three kinds of flavonoids using purified rat RGC cultures, and found that nicotiflorin and rutin had beneficial effects on hypoxic, glutamate, and oxidative stress at concentrations as low as 1 nM. The structure-activity relationships suggested that the sugar side chain of flavonoids might be important for neuroprotective activities. Moreover, rutin showed inhibitory effects on both apoptotic and necrotic pathways. Nicotiflorin and rutin have the potential to be promising neuroprotective drugs in the future.
